# The Use of Radium in Malignant and Non-Malignant Conditions with Report of Cases

**Published:** 1916-11

**Authors:** Gaston Torrance, W. C. Gewin, Walter A. Weed

**Affiliations:** Surgeon to Birmingham Infirmary; Surgeon to Birmingham Infirmary; Radiologist to Birmingham Infirmary, Birmingham, Ala.


### Journal-Record of Medicine

Successor to Atlanta Medical and Surgical Journal, Established 1855
and Southern Medical Record, Established 1870

**OWNED BY THE ATLANTA MEDICAL JOURNAL COMPANY**

PUBLISHED MONTHLY

*Official Organ Fulton County Medical Society, State Examing Board, Atlanta, Birmingham and Atlantic Railroad Surgeon's Association, Chattahoochee Valley Medical and Surgical Association, Etc.*

A. W. STIRLING, M. D., C. M., D. P. H.; J. S. HURT, B. Ph., M.D.
GEO. M. NILES, M. D., W. J. LOVE, M. D., (Ala.); Associate Editors
E. W. ALLEN, Business Manager

**COLLABORATORS**

W. F. WESTMORELAND, M. D., General Surgery
F. W. McRAE, M. D., Abdominal Surgery
ALLEN H. BUNCE, Medical Societies
E. B. BLOCK, M. D., Diseases of the Nervous System
MICHAEL HOKE, M. D., Orthopedic Surgery
CYRUS W. STRICKLER, M. D., Legal Medicine and Medical Legislation
E. C. DAVIS, A. B., M. D., Obstetrics
E. G. JONES, A. B., M. D., Gynecology
ALLEN H. BUNCE, M. D., Pathology and Bacteriology
R. T. DORSEY, JR., B. S., M. D., Medicine
L. M. GAINES, A. B., M. D., Internal Medicine
GEO. C. MIZELL, M. D., Diseases of the Stomach and Intestines
L. B. CLARKE, M. D., Pediatrics
EDGAR PAULLIN, M. D., Opsonic Medicine
THEODORE TOEPEL, M. D., Mechano Therapy
E. G. BALLENGER, M. D., Diseases of the Genito-Urinary Organs

VOL. LXIII. ATLANTA, GA, NOVEBMER, 1916 NO. 8

### THE USE OF RADIUM IN MALIGNANT AND NON-MALIGNANT CONDITIONS WITH REPORT OF CASES.

By Gaston Torrance, M. D., Surgeon to Birmingham Infirmary, W. C. Gewin, M. D., Surgeon to Birmingham Infirmary, and Walter A. Weed, M. D., Radiologist to Birmingham Infirmary, Birmingham, Ala.

Radium was discovered by P. and Madam Curie and G. Bemont in 1898. Its discovery was a sequel to H. Becquerel's observation in 1896 that uranium preparations emitted a radiation resembling x-rays, which had been discovered by Roentgen in 1895.

Some time after Becquerel's discovery Madam Curie made a systematic examination of the electric method of a large number of chemical elements and their compounds to test whether they possessed the "Radioactivities" or uranium.

In testing the activities of minerals containing uranium she found that the activity was always four or five times as great as that to be expected from their content of uranium and from this she concluded that there must be some other substance present more active than uranium. She was provided with a ton of the residues from the State manufactory at Joachimstahl Bohemia by the Austrian Government in order that she might carry out her experiments.

She separated a substance more active than uranium which she called polonium. A second substance was found to which the name of Radium was given—the name was well-chosen, as in the pure state radium bromide has a very great activity—about two million times as great as an equal weight of uranium.

Radium is continually losing matter and energy as electricity and heat, the temperature of radium salts is always a degree or two above the atmosphere.

The action of radium on the human tissues was not known until 1901 when Prof. Becquerel of Paris found, two weeks after carrying a tube of radium in his vest pocket, a severe inflammation known as the famous "Becquerel burn."

Active investigations have been carried on since this time to determine the action of radium on diseased tissues. In 1906 "The Laboratoire du Radium" was established in Paris.

Similar centers of study have been established in other countries since this time.

In using Radium externally it is necessary that the rays should be filtered to avoid superficial irritation and burns.

By this we mean that certain rays which we do not wish to use are arrested or absorbed by the interposition of certain substances or a given space. The Alpha rays are arrested by a sheet of letter paper or a distance equal to three centimeters.

Usually filters of lead, paper, rubber or mica are used for the alpha and soft beta rays and silver, aluminum, lead, brass and sometimes gold and platinum are used for the hard beta and the gamma rays.

**Physiological Action of Radium.** Douglas C. Moriarta (Med. Record, March 4, 1916), says it will often increase the white blood count to 250,000 in 48 hours, with rapid increase also of hemoglobin. It stimulates all cell life. Increasse elimination of carbon dioxide, urea and uric acid. Diminishes the viscosity of the blood and increases the secretion of urine, dilates the blood vessels and invariably lowers blood pressure.

**The Use of Radium in Non-Malignant Conditions.**

**Keloids**: Frank E. Simpson (Chicago), J. A. M. A., April 17, 1915, reports several cases completely cured; growth disappeared and all pain left, only small doses were used (six treatments of one hour each for eight days); in another case eight treatments of twenty minutes each extending over sixteen days were used.

He says pure keloids are more easily removed than those in which scar tissue is found, although these can be removed.

Hayward Pinch (Report London Radium Inst. 1914), London Lance, February 27, 1916, says good results are gotten, especially in recent and in young subjects. In tender and painful keloids the anesthetic effect of radium is generally marked and appears early in the treatment.

**Scars and Fibrous Conditions**: Laborde (Presse Medicale, August 5, 1915), reports a case of badly scarred forearm, with a neuritis of the median nerve, with disappearance of the scar and relief of the neuritis which was caused by pressure from the scar tissue. There was complete relief of the pain and restoration of the extensor function of the fingers.

**Trachoma**: Esdra (Policlinico, Rome), April 4, 1915, reports cures in all of twelve cases treated for trachoma.

**In the Treatment of Aural Diseases**: W. Sohier Bryant (Trans. Amer. Otological Society, 1914), reports his experience in the treatment of forty cases of chronic ear diseases; hearing was decreased in one case; unchanged in six cases; improved in 33; much improved in 14 cases; restored to normal in six cases.

**Exopthalmic Goiter:** Dr. Dawson Turner (Royal Infirmary, Edinburg), London Lancet, Feb. 27, 1916, reports five cases of exopthalmic goiter with marked improvement in all cases. The pulse rate dropped from 140 to 70 and the gland was not palpable in some of the cases after treatment.

**Arterio Sclerosis:** C. Everette Field (Med. Journal, January 22, 1916), advocates the use of radium in this condition and reports 190 cases with an average blood pressure of 190 and an average reduction of 40 mm.

Douglas C. Moriata (Med. Record, May 13, 1916), reports 56 cases treated during the past year and says there is invariably a drop of from 20 to 60 mm. in six weeks.

**Pernicious Anemia:** J. B. Bissell (Med. Record, June 19, 1915), reports a case of pernicious anemia cured by intravenous and intramuscular injections of radium solution, after having resisted all other forms of treatment.

**Splenomyelogenous Leukemia:** Renon, Degrais and Tournemelle (Bull. et Med. de la Med. des Hosp. de Paris, March 20, 1914), asserts that the results obtained by them with radium in the treatment of spleno-myelogenous leukemia in the last four years has proven it to be the most useful agent in their hands. The rapidity of the action of the remedy—the prompt subsidence of the splenic enlargement—the very pronounced decrease in the number of leucocytes and the marked improvement in the condition of the patients were striking features in the effects produced. One woman went through a normal pregnancy several months after having had the radium treatment and prior to that her condition had been very desperate. He reports twelve cases with marked effect on the spleen.

**Infections of Tissues and Bones:** J. B. Bissell (Med. Record, June 19, 1915), reports several cases of infections which cleared up promptly.

Dr. Wm. H. Cameron ,Pittsburgh) (Penna. Med. Jour., March, 1916), reports 46 cases of infection in English soldiers observed in the London hospitals during the present war. Most of the injuries were compound bone injuries and a number of them involved joints.

With the exception of four or five cases all were either cured or very much improved. There were two cases of gas bacillus infection, both of which were very much improved by only two or three applications of the radium. There was one case of osteomyelitis of the femur which had stood from October to the following August before having the bone removed, there were three sinuses discharging freely; all wounds healed up within thirty days with ten applications.

**Angio-Nerotic Oedema**: Hayward Pinch (Reports London Radium Inst., 1914, London Lancet, Feb. 27, 1915), reports three cases of angio-neurotic; one cured and two greatly benefitted.

**Arthritis Deformans**: Hayward Pinch (Reports London Radium Inst, 1914, London Lancet February 27, 1915), says very remarkable results have been gotten in some of these cases and especially when recent or peri-articular. Pain is relieved and an increase of mobility is noticed, which enables them to take care of themselves, shave, brush their hair and feed themselves, which very frequently they have been unable to do for years.

**Spastic Affections Spinal Cord After Gunshot or Shell Wounds**: G. Bonnus (Paris Medicale, Jan. 1, 1916), reports eleven cases. The best results were gotten in spastic hemiplegia from injury of the cord in the neck—but some benefit was realized in nearly every case. The injuries had been received from two to eleven months before. There had been no improvment for many weeks. Benefit was apparent from the first application. As the spastic phenomina subsided the pain from the inflammation of the roots became less or ceased entirely.

**Hemorrhage from Uterine Fibroids and Fibrous Conditions**: J. B. Bissell (Med. Record, June 19, 1915), reports the case of bleeding from an old lacerated fibrous cervix, which was completely relieved by the application of 75 mg. of radium for 18 hours.

Howard A. Kelly (J. A. M. A., Jan. 23, 1915), reports 36 cases from 30 to 67 years of age. This included pedunculated, submucous and interstitial growths. In all but one case the tumor either disappeared or was so much reduced in size as to give no further trouble. In every case of hemorrhage where intra uterine treatment was used the hemorrhage was controlled and wherever it was desirable amenorrhoea was produced. He considers it the ideal treatment in young women, where menstruation is sometimes preserved, and in very anemic cases.

Hayward Pinch (Radium Institute, London, 1914; London Lancet, February 27, 1915), says radium exerts a most beneficial action on menorrhagia, metrorhagia and usually reduces the size of the uterus and where there is obstinate leucorrhoea this, too, generally disappears.

**Radium in the Treatment of Malignant Conditions.**

**Sarcoma of the Long Bones:** Turner (British Med. Jour., Feb. 27, 1915), reporting the work done in the Royal Infirmary of Edinborough, 1914, gives one case of rapid growing sarcoma of the periostium of the lower end of the femur of a boy 13 years of age; the treatment caused the growth to shrink and become dense; it bled freely when the radium was first introduced and two months later there was scarcely any bleeding and it was so dense that the introduction of a tube of radium was scarcely possible.

**Lymphangioma:** Robt. Abbe (Med. Record, Aug. 7, 1915), reports six cases, in which two were of the neck, three of the tongue and one of the leg with excellent results.

Frank E. Simpson, Chicago (J. A. M. A., Mar. 25, 1916), reports a case in which the whole right buttocks was involved of 15 or 25 years' standing. A number of short exposures (1 hr.) were made. A photograph taken 18 months later showed a smooth perfectly healed surface. He concludes that radium is the method of election in the treatment of these cases.

**Lymphosarcoma**: Kelly and Burnam (J. A. M. A., Jan. 15, 1916), report a series of twenty cases, most of them in a very advanced stage. Twelve had been operated upon with rapid recurring masses of the neck. Thirteen of the twenty (65 per cent.) are apparently cured. They say "from our experience lymphosarcoma ought not to be operated upon but should be subjected to radium therapy at the earliest possible moment." Several of these cases showed primary extension to the mediastinum and these responded as readily as those more superficially placed.

**Skin Cancer**: Reports from the London Radium Institute for 1913 show that out of 181 cases of cancer of the face, neck and breast 154 healed; 7 not healed; 3 under treatment; 7 discontinued.

They quote from the work done in 1910, one hundred and fifty-four healed cases and state that of this number 135 remained healed in 1914.

**Cancer of the Mouth**: Anton Sticker (Berliner klinische woshen, October 4, 1915), reports 15 cases, in most of which the malignant disease retrogressed without scar or mutilation. Three cases of cancer of the tongue were cured. Seven of the cases were of the lower and five of the upper jaw. In two cases the cancer was a recurrence after several operations. He holds that radium is preferable to surgery in many cases for technical reasons as well as from the standpoint of immunity. Under radio therapy the cancel cells being gradually destroyed and passing into the circulation cause a lively production of antibodies and these protecting substances combat the growth of any new cancer cells.

**Cancer of the Bladder**: Hayward Pinch (London Radium Inst., London Lancet, February 27, 1915), reports six cases with most gratifying results, the disappearance of fungating growths, the arrest of hemorrhage and discharge, healing of ulceration and relief from pain are phenomena of frequent occurrence.

W. Ayers (Surgery Gyn. and Obs., Sept., 1915), reports a case of bladder cancer in a man 75 years of age treated by direct application of the radium through a cystoscope, with complete disappearance of the cancer.

**Cancer of the Rectum:** Pellizzari (Riforma Medica, March 20, 1915), reports a case in which the rectum was almost obstructed; very adherent; inoperable. The case was treated in 1913 and again in 1914, soon regained strength and weight. Some bulging of the rectum remained but the malignant growth was completely removed.

Hayward Pinch (Radium Inst., London, 1914, Lancet, February 27, 1915), says the soft annular and vascular type are much more favorably affected than the hard non-annular type with subjacent adhesions, and that those in the upper part of the rectum respond more readily than those lower down.

Henry Schmitz (Surgery Gyn. and Obs., March, 1915), reports three cases of cancer of the rectum with apparent cures in two cases and the third refused to continue the treatments on account of some rectal tenesmus.

**Cancer of the Uterus and Vagina:** Pozzi and Rouhier (Revue de Gyn. et de Chir. Abdom., Paris, June, 1915), reports six cases from Pozzi's service in which hysterectomy plus radium was used. They think vaginal hysterectomy with the radium treatment gives much better results than the more extensive abdominal operations.

B. H. Boggs (Amer. Jour. Roentgenol, 1916, Vol. 3-92), reports 14 cases treated by radium and followed by the x-ray for inoperable uterine cancer. Three have been clinically cured. All were much improved but two. In two others the disease had almost disappeared when his report was made.

E. von Seuffert (Zentralb 1. f. d. ges. Gyak. u. Beburtsh. s. d. Grenzge, 1914, Vol. IV, 740), reports 54 cases from Doederlein's clinic of uterine cancer whose treatment can now be regarded as successfully completed. He says these patients are well and are working just as they did before the disease began.

He considers the results secured by this method are far in excess of the results secured by any other method.

**Cheron and Rubens-Duval** (Strahlen Therap. 1914, vol. V-1), during the past five years have studied more than 150 cases clinically and histologically. They observed one case of recurrence that had remained well for two years after having been treated with radium. They say that it is generally accepted that complete retrogression lasting more than a year, in the majority of instances, is a complete cure. One hundred and fifty-eight cases are reviewed, all of which, practically, were inoperable, with complete retrogression in 77 cases; 46 showed no recurrence, of which 22 had been free for more than a year; 31 showed only local recurrence which were cured later by further treatments; 12 cases became operable. In only two cases was no improvement shown.

**Polubinsky**: (Russkiy Vrach, Petrograd, Jan. 3, 1915), reports 23 cases; there were nine complete recoveries, 7 of which were inoperable; of the other 14 cases six relapsed after some improvement and the others were lost sight of or gave doubtful results.

**Kelly and Burnum**: (J. A. M. A., June 17, 1916), report 327 cases, this includes borderline cases, those with cancer fixed to the pelvic wall, great massive cancers choking the pelvis and many with general metastases in which the radium was only used for the relief of pain. Over 20 per cent. are apparently cured. In a large number not cured the discharge stopped, the pain ceased and the patient's health was built up.

**Hayward Pinch**: (Report London Radium Inst., 1914; Lancet, February 27, 1915), says uterine cancer continues to yield most gratifying results, and the effects of radium treatment in inoperable cases are far in advance of those obtained by any other known medical or surgical method.

**Henry Schmitz**: (Surgery Gyn. and Obs., March, 1915), reports twenty cases from A. J. Ochner's Clinic; practically all of the cases were inoperable, but were much improved for about six months and some later died from recurrences. Six of his cases were too recent to report as cured. He says: "I am inclined to the firm belief that radium possesses a remarkable therapeutic action and will become an indispensable adjunct in the routine treatment not only of cancer but also of a great number of other diseases.

**S. Flatau!** (Zentralblatt fur Gyn., Leipzig, Aug. 28, 1915), says he has not operated for cancer of the cervix since 1913 and his proportionate number of women living and cured under radio-therapy alone is larger than he could ever report before with abdominal operations for cancer of the cervix. He says further, it is now beyond question that an incipient cancer focus can be destroyed by radium without leaving a trace.

**P. Degrais** (Annals de Gynecologie et d'Obstetrique, Paris, Sept., 1915), suggest "Curie Therapie" for the treatment with radium as radiotherapy is indiscriminately used for various forms of radiant energy in therapeutics. He has a number of cases who seem to be in perfect health two, three and four years after cross-fire radium treatment for cancer.

**Kelly and Burnam:** (J. A. M. A., Nov. 27, 1915), report 213 cases of cancer, 14 were operative cases and the others were inoperative, 203 were treated by radium alone, 57 of these cases were cured and traced; one for 6 years; 3 for 4 years; 4 for 3 years; 5 for 2 years; 29 for one year; 15 for over six months.

**C. Jeff Miller,** New Orleans: (Surg. Gyn. and Obs., April, 1916), reports 26 cases that were inoperable or had recurred; 15 inoperable cases had radium alone, of these five cases are apparently cured. He concludes "that the results so far are superior to any other method I have tried in similar cases."

**R. St. Clair:** (West. Med. Times, Vol. 35, 1915), reports three cases of malignancy, two of the uterus and one sarcoma of the leg in which all of the diseased tissue could not be removed, which were treated by radium, all apparently cured—one case for 14 months.

**Fabre:** (Ann. de Gynecol. et d'Obst., Vol. xl-620, 1915), gives histories of ten cases in which she used radium with excellent results. In borderline cases she thinks the radium should be used before and after operation.

**Immunity (Cancer):** A Clifford Morson, London (Br. Med. Jour., Vol. II, 1915), says experiments with mice show the cancer cell has the power to confer immunity against the same tumer if irradiated with a sublethal dose but this immunity process is lost if the cells receive a lethal dose.

Dr. W. M. Crofton (Royal Academy Med. Ireland, London Lancet, February 27, 1915), says that radium not only produces a local effect on the cancer but there was also a general reaction and suggests that this is due in part to the absorption of cancer cells into the circulation, thereby producing a certain amount of immunity.

**Can Cancer Cells be Destroyed by Radium?** Cheron and Rubens-Duval (J. A. M. A., Noverber 27, 1915), treated a patient suffering from inoperable cancer of the cervix with radium during November, 1910, and January, 1911; she died of an intercurrent disease 15 months later. All of the internal organs and tissues were removed postmortem and a careful sereal histologic examination made of all the tissues did not reveal a single carcinoma cell.

**J. B. Bissell:** (Surgery Gyn. and Obs., July, 1915), reports eleven cases of carcinoma and sarcoma, in which pathological examinations were made, that the malignant growths disappeared and remained so up to the time his report was made.

He quotes Gauss' description of the ten pieces of tissue excised at various times while under radium treatment; the first section taken showed adenocarcinoma; a section taken from the same place two weeks later connective tissue and cancer nests; three weeks later the cancer evidences have disappeared and the miscroscope showed nothing but benign tissue with epitelial debris.

**Sir Thos. Oliver:** (New-Castle-upon-Tyne; London Lancet, February 6, 1915), reports the case of a widow of 62 with recurrent cancer of the vagina and uterus involving the bladder and rectum, in the microscopic examination showed cancer. The case was considered hopeless and as a last resort radium was tried. Six weeks later the growth was much smaller; four months after the treatment was begun the growth had entirely disappeared. Vaginal mucous membrane and septum between vagina and rectum seemed to be perfectly normal. Her bladder and rectal functions are normal, she has put on considerable weight, looks many years younger and looks the perfect picture of health.

### Resume of Cases.

**Epitheliomata.** Of sixteen cases treated, six are apparently cured, four improved, two not improved, two, in which it is too early to note results, one improved, but has not returned for further treatment, and one died.

The treatment of epitheliomata by means of radium has, on the whole, been very gratifying. Cases, in which only the cutaneous surface is involved, has responded promptly.

Epitheliomata of the cutaneous surface, if flat and superficial, and not accompanied by ulceration are given from 20 to 100 Mg. hours, using a flat half strength applicator, with no screening except one thickness of pure rubber dam. If the cosmetic effect is to be considered, screening up to 1 Mm. of silver may be used with a corresponding increase in the length of exposure.

Where there is much infiltration it is necessary to use the tubes of radium in order to increase the intensity and depth of exposure. The deeper the infiltration, the more screening necessary with a prolonged exposure. Often as much as 100 mg. is used screened with 1 Mm. of silver and applied for 24 hours. This is given in four to six hours exposure extending over a period of four to six days.

Epitheliomata of the mucous surface are much more obstinate than when only the skin is affected. Where the growth is near a muco-cutaneous junction it is desirable to give radium treatment before it reaches the mucous surface, as a much more favorable prognosis may be given.

Melanotic epitheliomata, as well as melonotic sarcoma, are apparently but little influenced by radium. We have had only one case of this type, which was operated upon by the Drs. Mayo of Rochester with a most unfavorable prognosis. At the time radium treatment was begun there were metastases in the chest. The progress of the disease was not checked, but the pain was greatly relieved by the radium applications.

Six of the cases had been treated previously by the x-ray. Of these, three are apparently cured, one much improved, one, unimproved, and one, in which insufficient time has elapsed to note results.

The cases that have shown no improvement were very unfavorable to begin with because of the advanced stage of the disease, or inaccessibility of the growth.

**Carcinomata of the Breast.** Of five cases treated radium was used in four of them immediately after operation as a prophylactic measure against recurrence. The other case was one of recurrence two years after operation. She was in a hopeless condition when first seen, and was advised that the treatment would only be palliative.

All operable cases of cancer of the breast should be operated upon before using radium. The progress of inoperable cases is much retarded, and the pain lessened.

**Carcinoma of the Uterus.** Of four cases treated, two are apparently cured, one improved, and one the results not known, as she did not return for further treatment.

The treatment of carcinoma of the uterus with radium has been very satisfactory. Of the two cases that are apparently cured one had a fungating growth of the cervix, which completely filled the upper part of the vagina. This case was first treated by placing 200 mg. of radium element, well screened, in the vagina in close proximity to the growth. The growth was completely gone in about six weeks. 100 Mg. of radium was then placed in the cavity of the uterus and left for five hours on three successive days, making a total exposure of 1500 Mg. hours. There are no further symptoms and the patient is gaining in weight.

The other case was one of the "Excavating type" of carcinoma of the cervix involving the body of the uterus. The tumor has been cauterized by Dr. Baker of Charleston, S. C., who pronounced it inoperable and advised her to seek radium treatment.

At the time she came for treatment the cervix was entirely gone; the uterus was completely fixed; there was considerable hemorrhage, and a very profuse discharge. Since treatment there is no hemorrhage, or discharge, and she is in excellent health.

The case that is designated as "improved" was one in which the uterus, the cervix, and broad ligaments all were involved, and was in a very advanced stage when first seen. No hope was entertained from the beginning so far as a cure was concerned, but it has been surprising how much improvement has taken place. The subsidence of the pain and offensive discharge has added very materially to her comfort. Also, no doubt, her life will be considerably prolonged.

The other patient was almost in a moribund condition when first seen, and was advised that treatment at best would be only palliative. We have not heard from her since she left the hospital.

**Carcinoma of the Rectum.** Of two cases treated one was too far advanced to expect permanent improvement. The uterus and adjacent tissues were a large cancerous mass. The rectum also, was extensively involved. A colostomy was done in March, 1916, by Dr. C. C. Cody, of Houston, Texas, which was performing its function admirably. Radium treatment was given in June, 1916. We have not heard from her since treatment.

The other case is still under treatment. Since beginning the radium applications the pain has almost completely subsided, the bowel derangement is very much lessened, and on the whole, the patient is much more comfortable.

As this patient is an elderly lady, and is already cachetic, what we hope to do is to control the pain, keep the bowel patent, and thus avoid the disagreeableness of a colostomy.

**Carcinoma of the Bladder.** The only case of carcinoma of the bladder treated was a secondary condition following cancer of the uterus, which was referred by Dr. O. P. Board, of Birmingham. This patient had a total hysterectomy at St. Thomas' Hospital, Nashville, for carcinoma of the uterus in November, 1914. She remained apparently well until February, 1916, when she noticed blood and shreds in the urine. The bleeding was profuse at times. When seen in April, 1916, cystoscopic examination revealed a rather extensive involvement of the bladder about the trigone. Radium treatment was given by introducing tubes of radium into the bladder. She returned for treatment in June following; at this time the symptoms had entirely disappeared, and cystoscopic examination showed a very decided improvement of the local condition.

**Paget's Disease of the Breast.** In one case treated the superficial lesion was entirely cured by the first treatment. The nodule in the breast, which was about the size of a hen-egg, was materially lessened; and a letter from her states that it is still decreasing in size since the second treatment.

**Sarcoma of Breast.** The relief in one case treated was only palliative, as there were metastases of the long bone, and mediastinum when she came for treatment.

**Carcoma of Parotid Gland.** We have had two cases which were both in an advanced stage when first seen. One case had been operated upon for tumor of the breast (supposedly sarcoma) about two years ago. At the time treatment was given there were two growths about the size of large marbles near the original breast incision, one nodule about the same size on top of the head, and a large tumor of the parotid gland; and, also, probable involvement of the mediastinum. The redium made her much more comfortable but did not check the progress of the disease.

The other case has been under observation but a short time. No definite results have been noticed.

**Sarcoma of the Intestine.** Of two cases treated one has been under treatment but a short time. She was operated upon in May, 1915, by Deaver, of Philadelphia, for a retro-peritoneal tumor which proved to be a fibro-sarcoma. She has a rather large recurrent intra-abdominal mass. Since beginning radium treatment the pain has subsided and on the whole, the patient is feeling much better. Insufficient time has elapsed to expect a marked retrogression of tumor.

The other case was referred by Dr. W. P. McAdory, of Birmingham, who, on February 12, 1916, removed a tumor of the ilium and mesentery, about three feet of intestine was excised. Pathological examination showed the tumor to be sarcoma. About July 1st, he was referred by Dr. McAdory for radium treatment. At that time there was a palpable tumor apparently as large as a man's fist. Also he had diarrhoea, and a very foul odor of breath. At times he suffered considerable pain. Treatment was given from July 7th, to 13th, inclusive.

He returned for second treatment August 10th. Patient's condition was much improved; the foul odor of breath had entirely disappeared, bowels were normal, suffered very little pain, and the growth was appreciably smaller.

**Goitre.** Two goitres have been treated, one exophthalmic, and one simple.

The exopthalmic goitre improved rapidly. The tachycardia gradually subsided, the gland reduced in size, and the nervousness disappeared.

**Hodgkin's Disease.** Only one case has been treated which was referred by Dr. Gaston Greil, of Montgomery. Dr. Greil operated on her in July, 1914, removing glands of right side of neck.

Dr. Greil referred her for radium treatment on June 27, 1916. At that time she was thin and emaciated. There was enlargement of the glands near the angle of the right lower maxilla, also of left supra-clavicular glands.

After giving radium treatment she seemed to improve for a few weeks. Since then a letter states that she has lapsed back to about the same condition as she was in when treatment was given.

**Fibroids of Uterus.** Three cases of fibroids of the uterus have been treated, all of which are clinically cured.

The treatment of fibroids, when not too large and accompanied by menorrhagia or metrorhagia is highly satisfactory.

The cases treated have responded promptly. The discharge and hemorrhage has entirely stopped and the fibroids have decreased in size.

